# Supplementary figures and images for: Years of life lost to COVID-19 in 49 countries: A gender- and life cycle-based analysis of the first two years of the pandemic
Source: PLOS Glob Public Health. 2023 Sep 18;3(9):e0002172. doi: 10.1371/journal.pgph.0002172 (PMC10506703; doi:10.1371/journal.pgph.0002172)

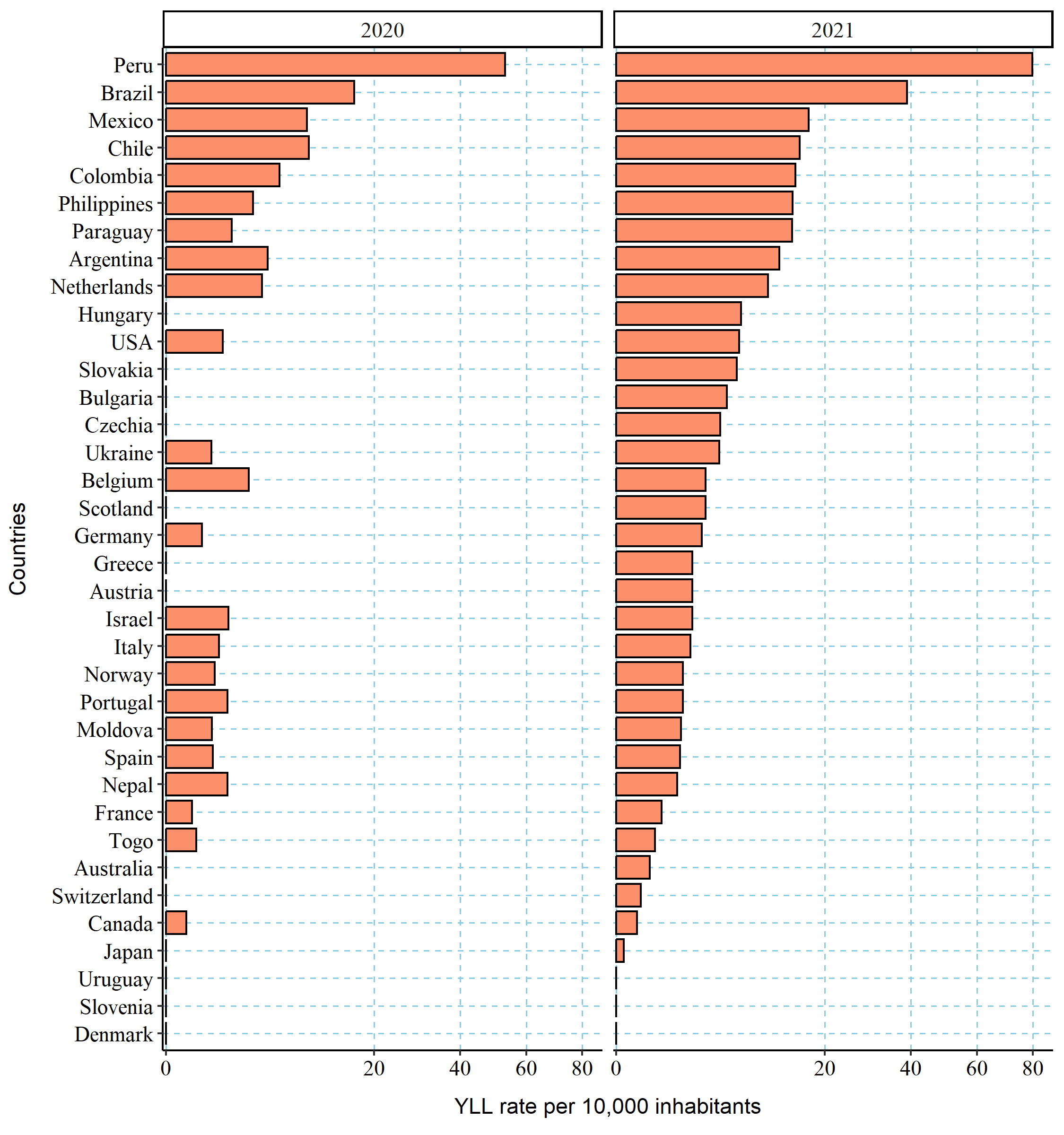

Supplement: S1 Fig — (TIFF) [file pgph.0002172.s001.tiff]

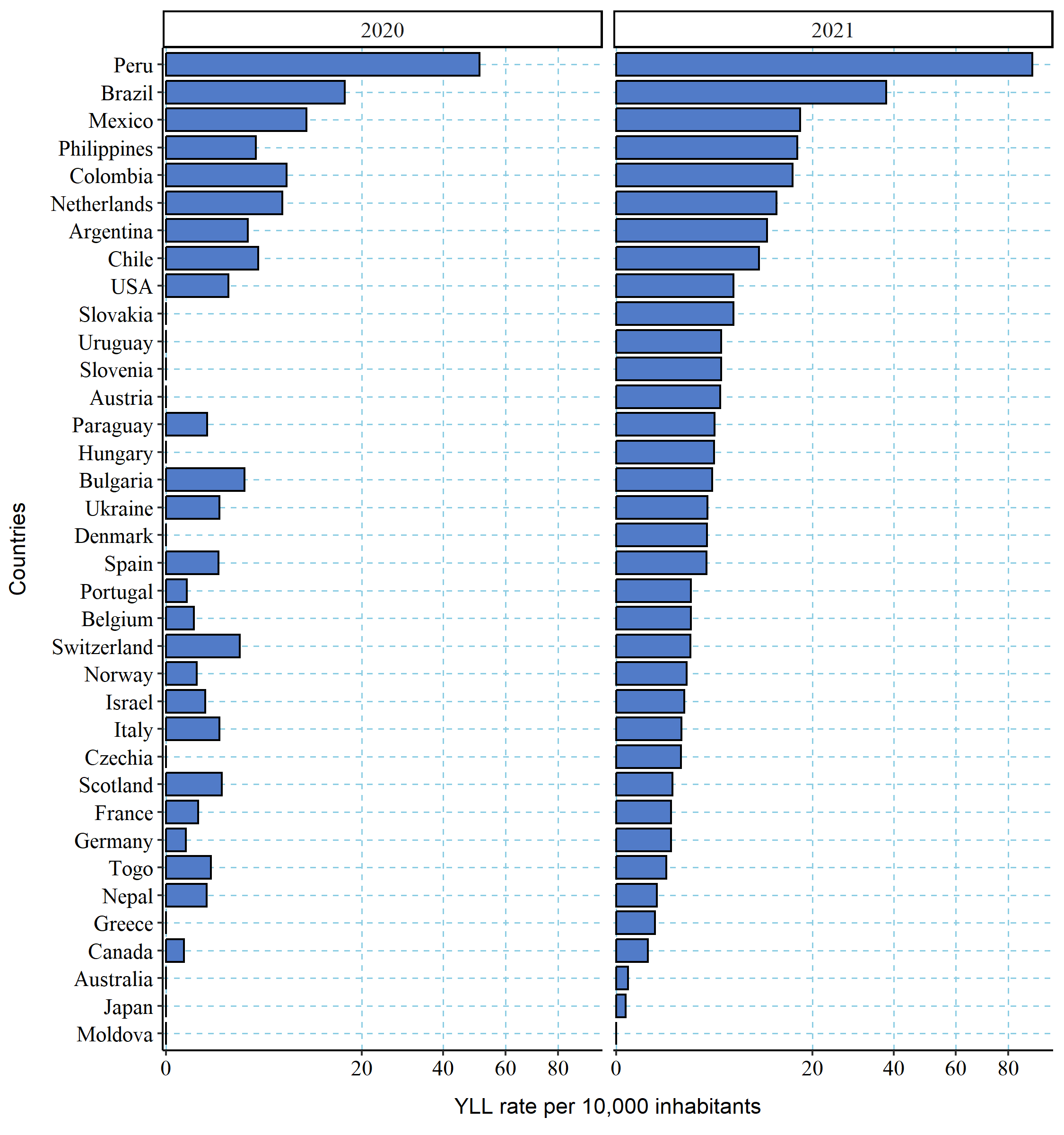

Supplement: S2 Fig — (TIFF) [file pgph.0002172.s002.tiff]

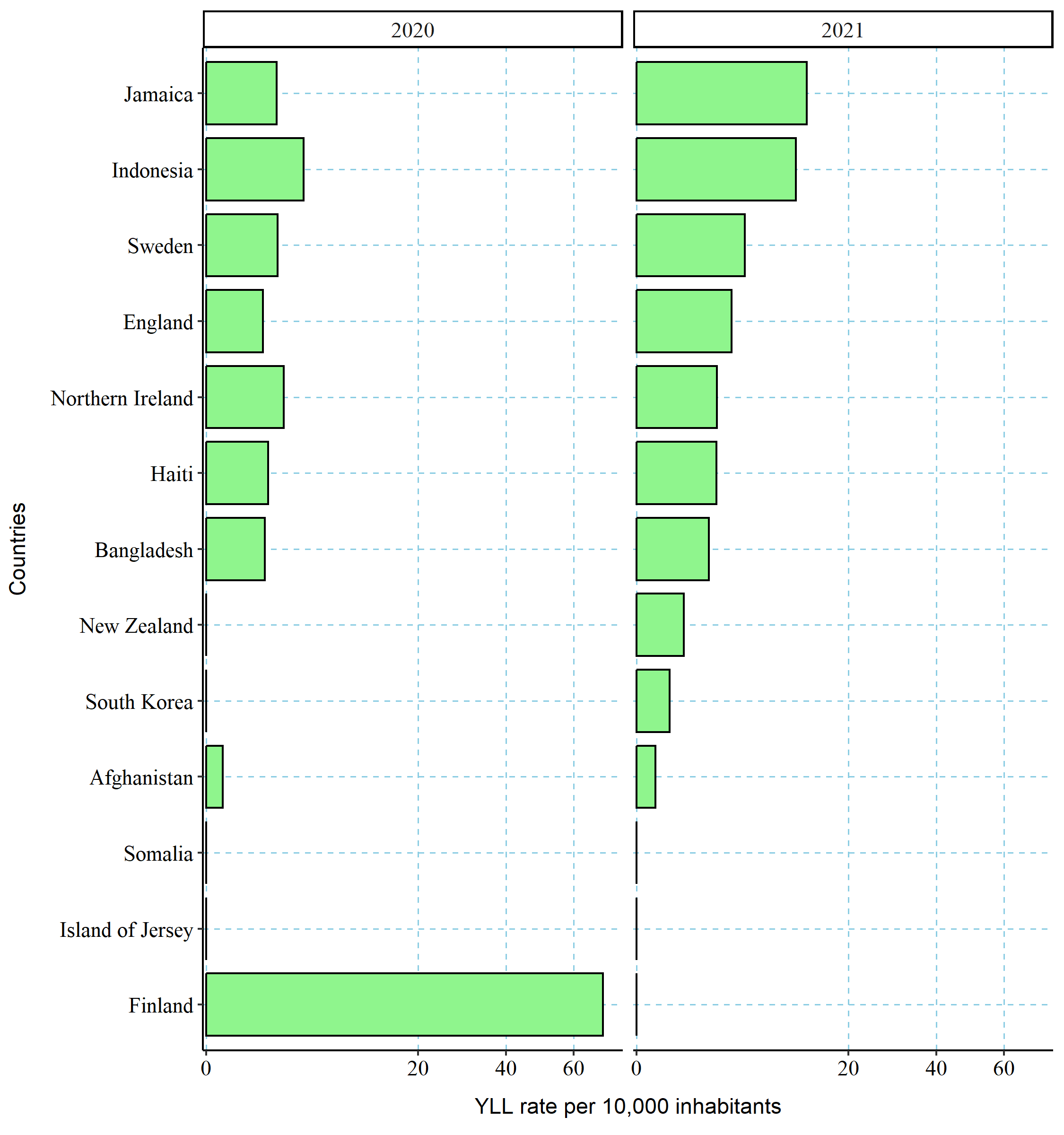

Supplement: S3 Fig — (TIFF) [file pgph.0002172.s003.tiff]

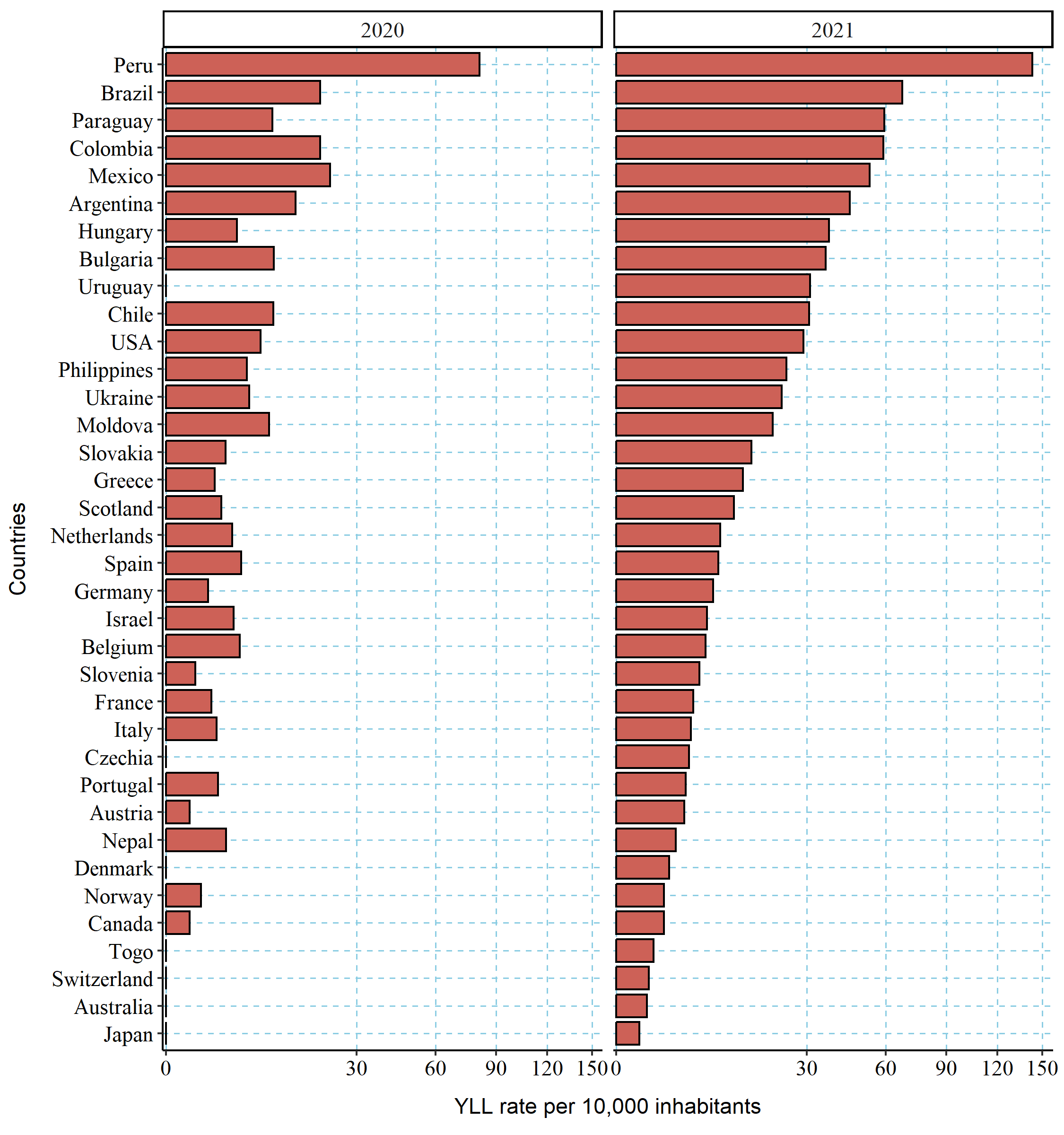

Supplement: S4 Fig — (TIFF) [file pgph.0002172.s004.tiff]

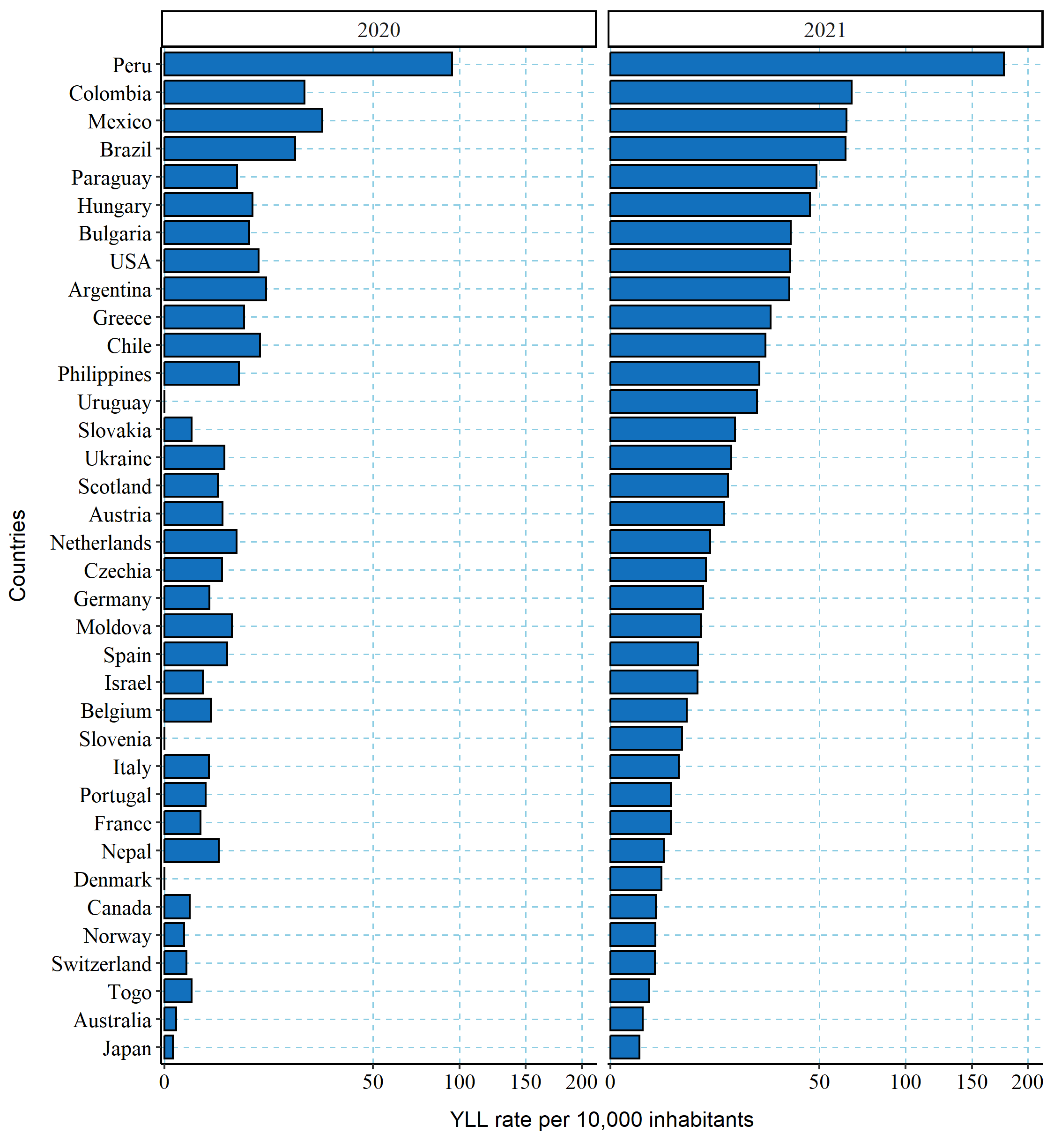

Supplement: S5 Fig — (TIFF) [file pgph.0002172.s005.tiff]

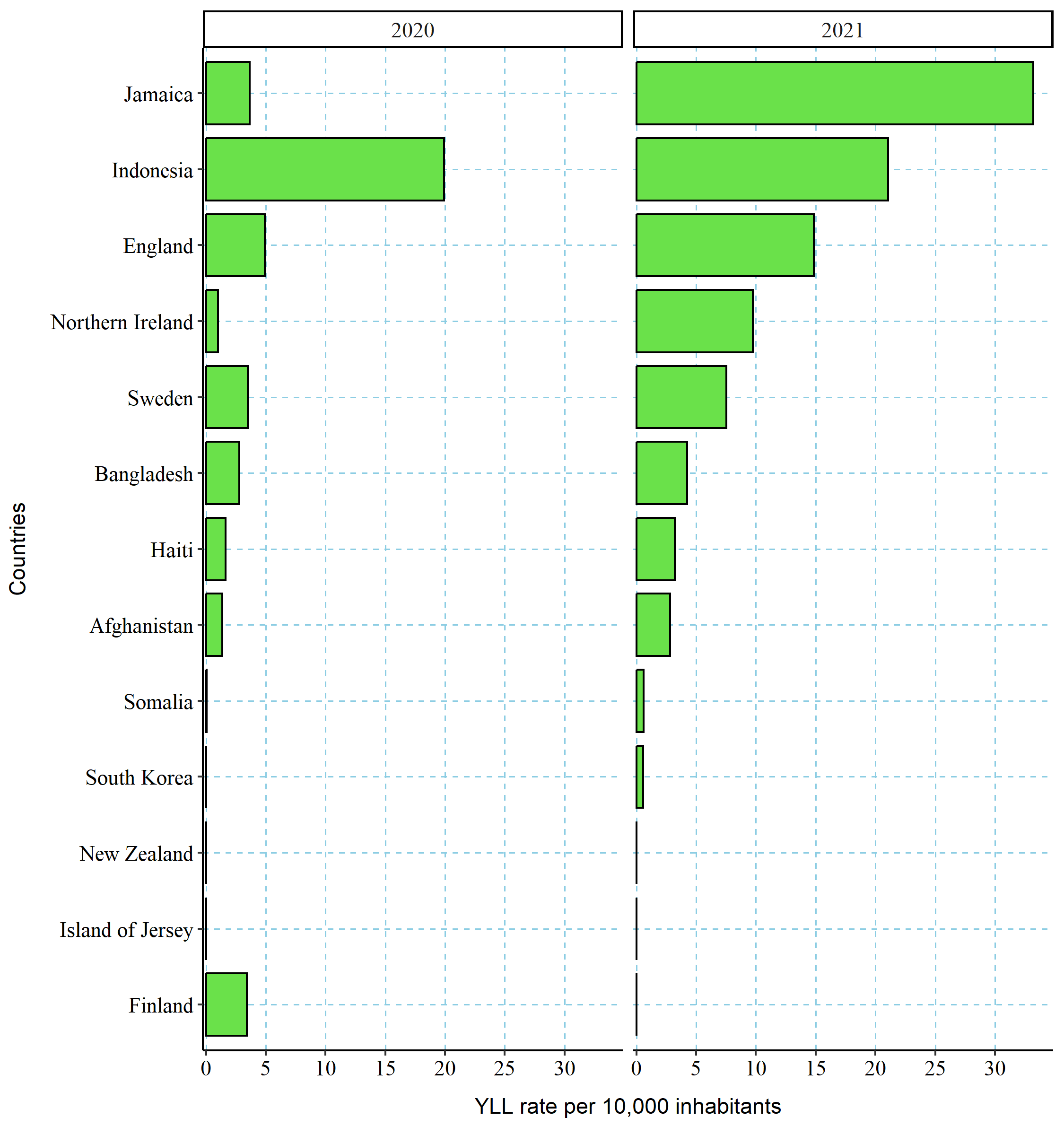

Supplement: S6 Fig — (TIFF) [file pgph.0002172.s006.tiff]

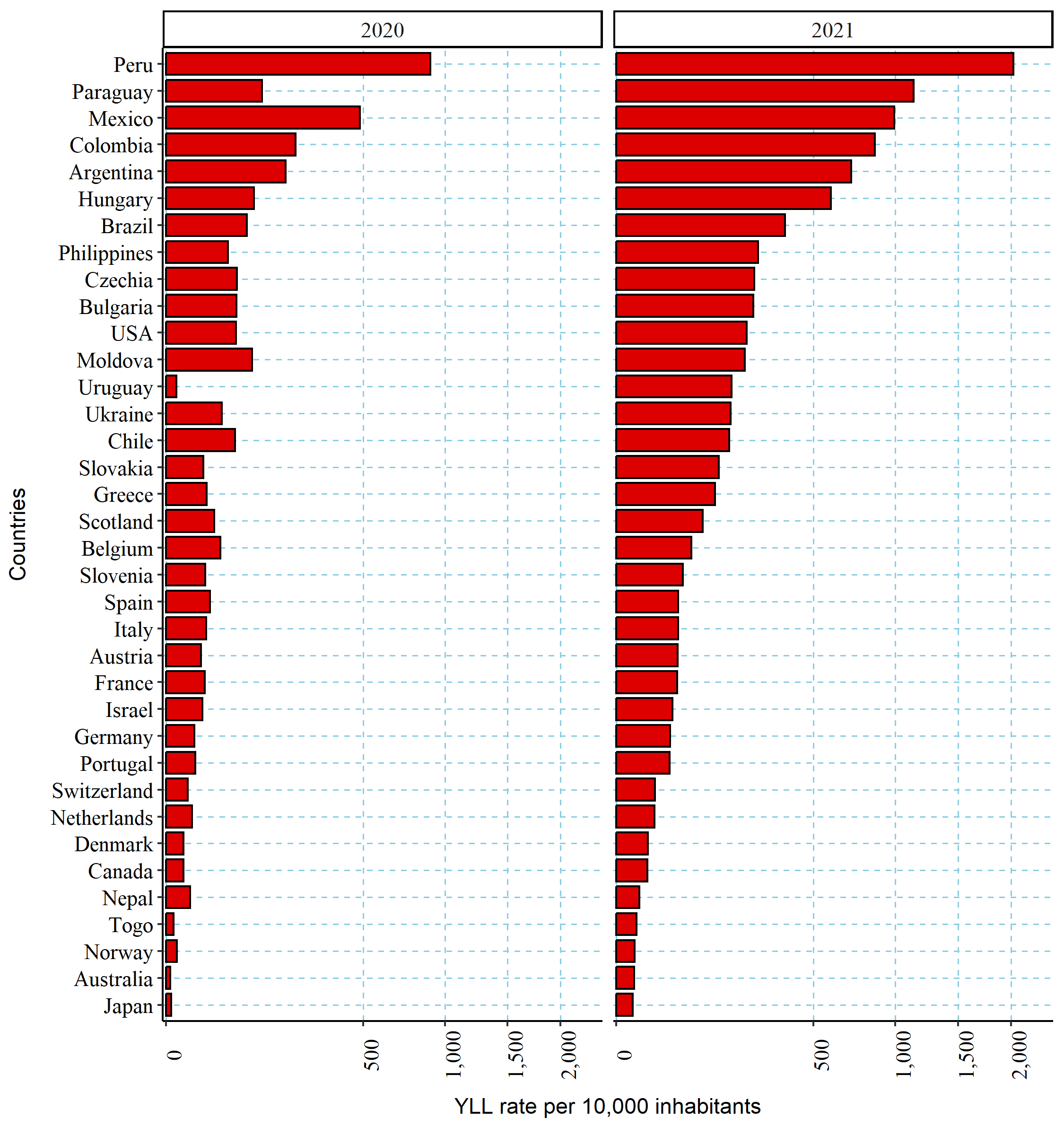

Supplement: S7 Fig — (TIFF) [file pgph.0002172.s007.tiff]

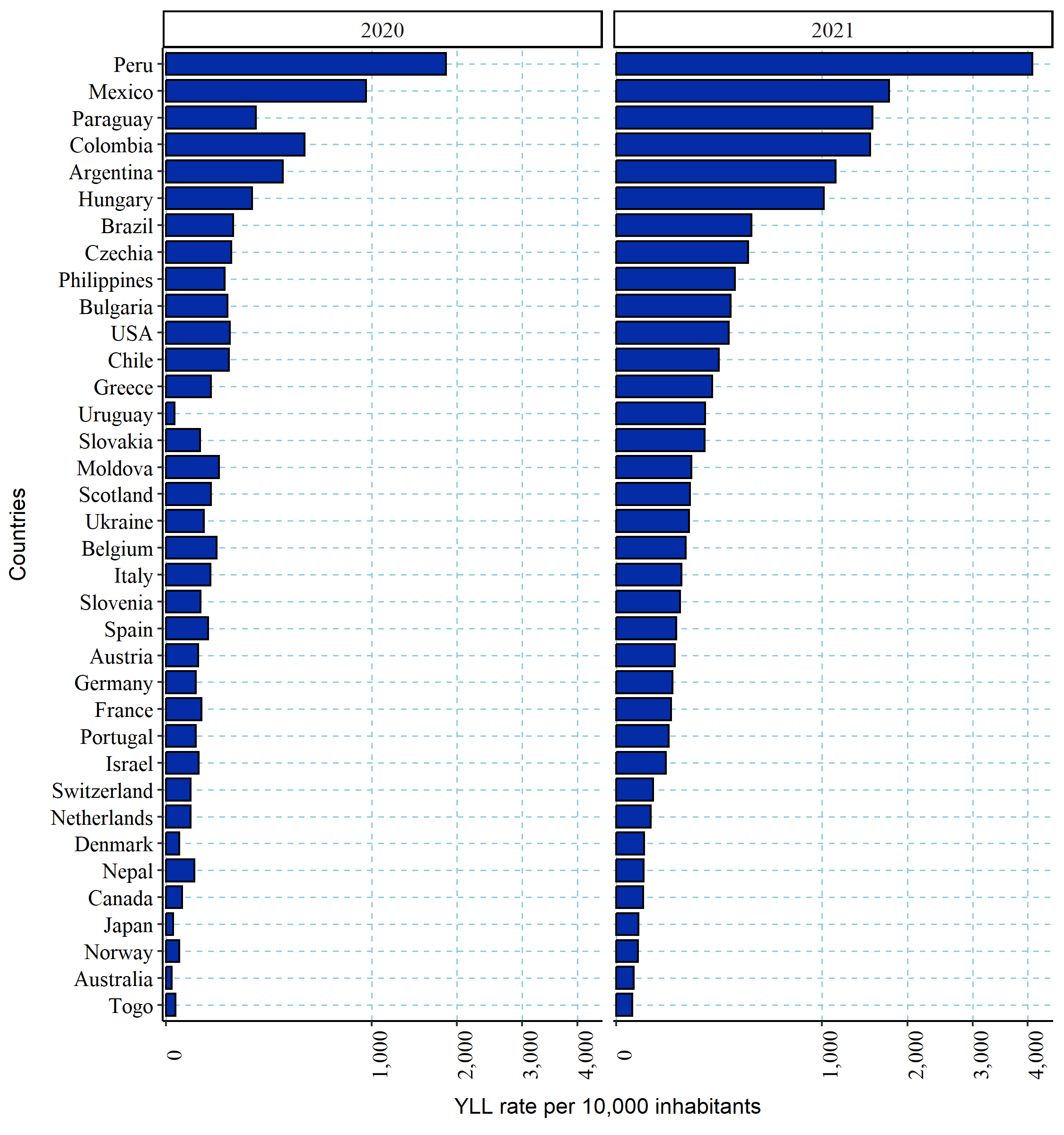

Supplement: S8 Fig — (TIFF) [file pgph.0002172.s008.tiff]

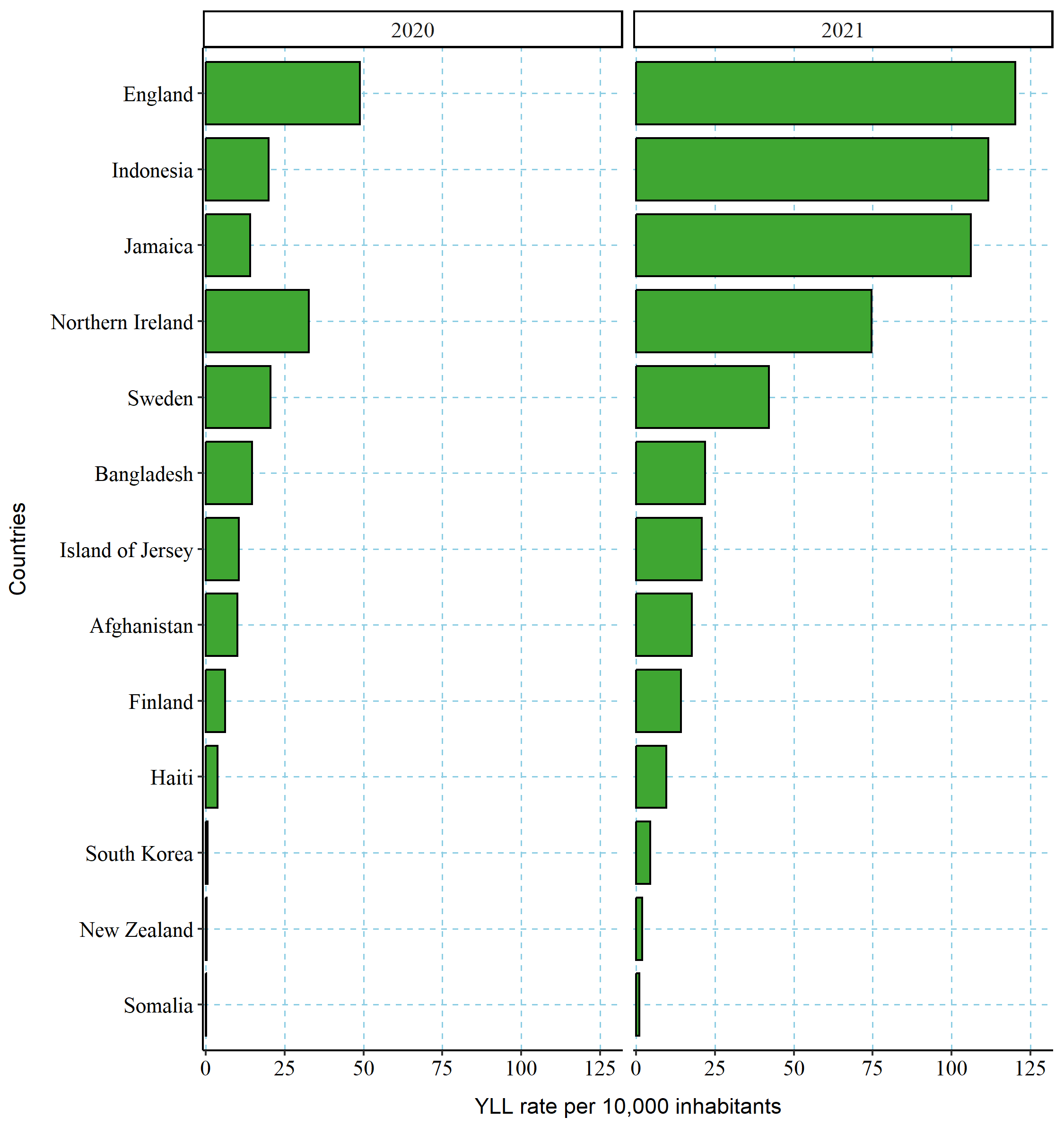

Supplement: S9 Fig — (TIFF) [file pgph.0002172.s009.tiff]

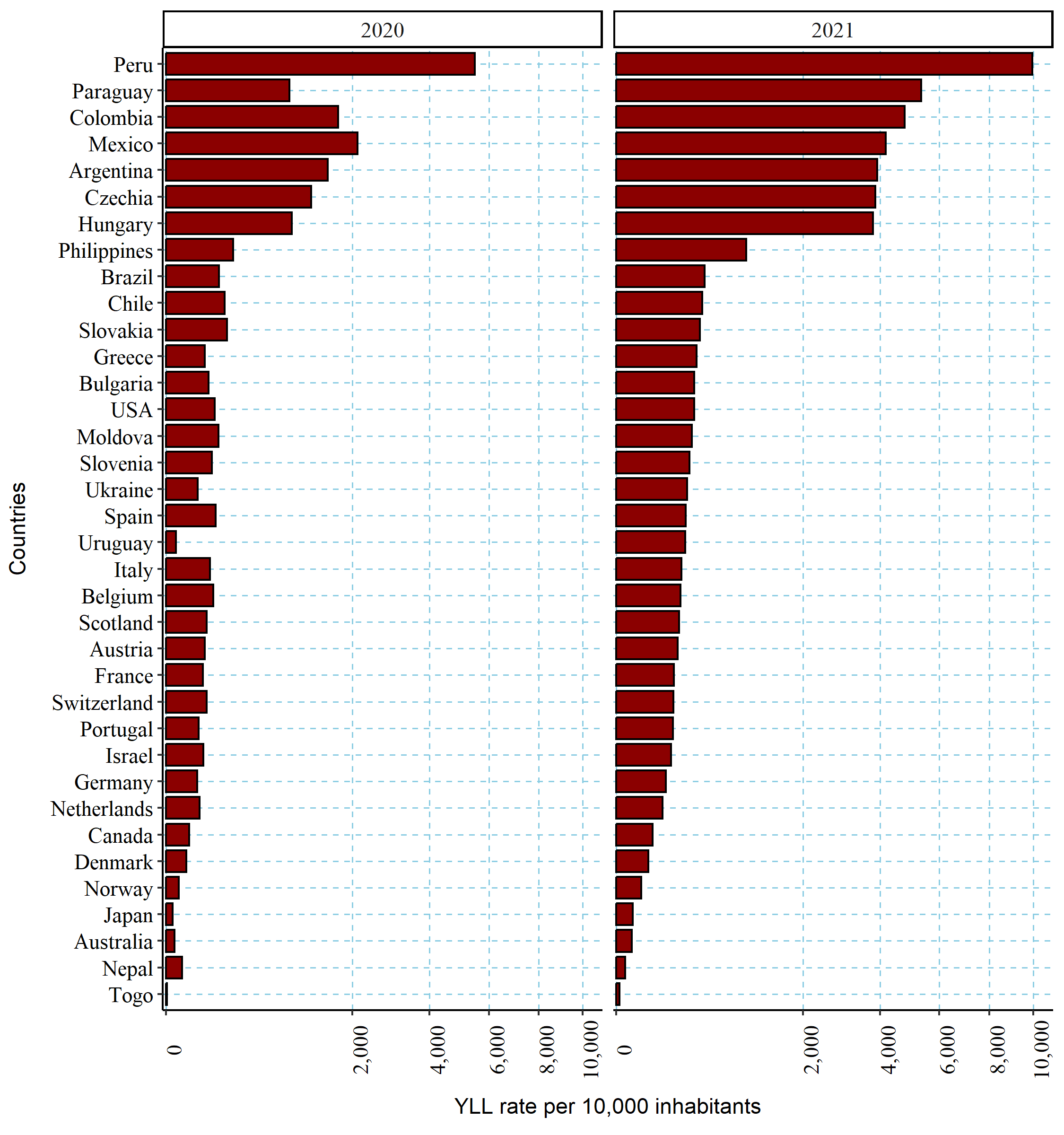

Supplement: S10 Fig — (TIFF) [file pgph.0002172.s010.tiff]

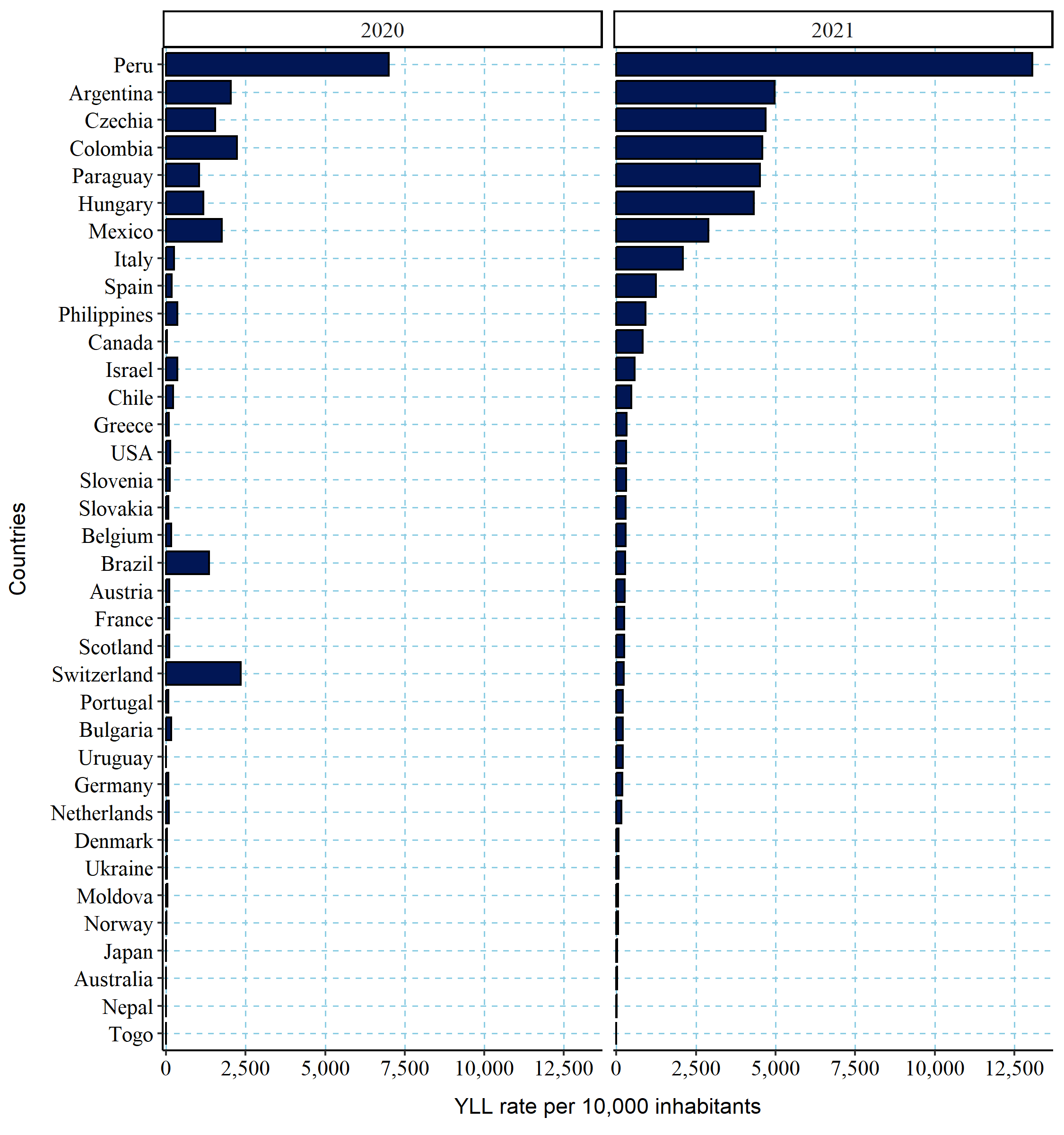

Supplement: S11 Fig — (TIFF) [file pgph.0002172.s011.tiff]

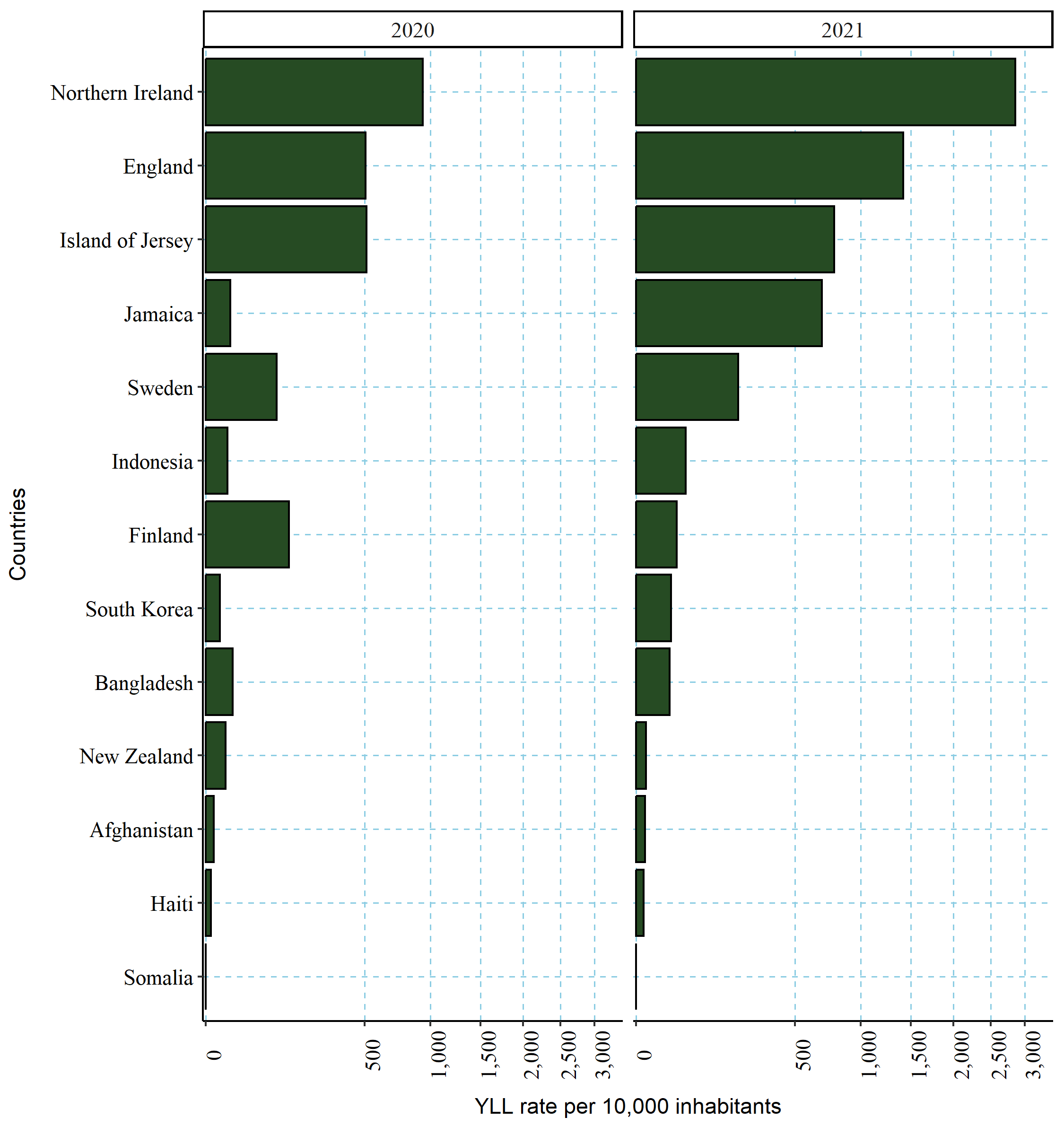

Supplement: S12 Fig — (TIFF) [file pgph.0002172.s012.tiff]
