# Supplementary material for: Years of life lost to COVID-19 in 49 countries: A gender- and life cycle-based analysis of the first two years of the pandemic
Source: PLOS Glob Public Health. 2023 Sep 18;3(9):e0002172. doi: 10.1371/journal.pgph.0002172 (PMC10506703; doi:10.1371/journal.pgph.0002172)
Supplement: Attachment — Submitted filename: R&R comments to reviewers.docx [file pgph.0002172.s018.docx]

**Response to reviewers**

**Manuscript Title: Years of life lost to COVID-19 in 49 countries: A gender- and life cycle-based analysis of the first two years of the pandemic.**

**Reviewer(s)' Comments to Author:**

Journal Requirements:

1. Please provide a copy edited of your paper.

**Answer:** We greatly appreciate all the comments provided in the article; they were highly productive. Thanks to your suggestions, we believe that the revised version of the article has significantly improved in quality. We are providing a revised copy of our paper, both in a clean format and with tracked changes. Additionally, we have made the necessary adjustments to meet the journal's formatting requirements.

2. Some material included in your submission may be copyrighted. According to PLOS’s copyright policy, authors who use figures or other material (e.g., graphics, clipart, maps) from another author or copyright holder must demonstrate or obtain permission to publish this material under the Creative Commons Attribution 4.0 International (CC BY 4.0) License used by PLOS journals. Please closely review the details of PLOS’s copyright requirements here: PLOS Licenses and Copyright. If you need to request permissions from a copyright holder, you may use PLOS's Copyright Content Permission form.

Please respond directly to this email or email the journal office and provide any known details concerning your material's license terms and permissions required for reuse, even if you have not yet obtained copyright permissions or are unsure of your material's copyright compatibility.

Potential Copyright Issues:

Figure 1: please (a) provide a direct link to the base layer of the map (i.e., the country or region border shape) and ensure this is also included in the figure legend; and (b) provide a link to the terms of use / license information for the base layer image or shapefile. We cannot publish proprietary or copyrighted maps (e.g. Google Maps, Mapquest) and the terms of use for your map base layer must be compatible with our CC-BY 4.0 license.

Note: if you created the map in a software program like R or ArcGIS, please locate and indicate the source of the basemap shapefile onto which data has been plotted.

If your map was obtained from a copyrighted source, please amend the figure so that the base map used is from an openly available source. Alternatively, please provide explicit written permission from the copyright holder granting you the right to publish the material under our CC-BY 4.0 license.

Please note that the following CC BY licenses are compatible with PLOS license: CC BY 4.0, CC BY 2.0 and CC BY 3.0, meanwhile such licenses as CC BY-ND 3.0 and others are not compatible due to additional restrictions. If you are unsure whether you can use a map or not, please do reach out and we will be able to help you. The following websites are good examples of where you can source open access or public domain maps:

* U.S. Geological Survey (USGS) - All maps are in the public domain. ([http://www.usgs.gov](http://www.usgs.gov/))
* PlaniGlobe - All maps are published under a Creative Commons license so please cite “PlaniGlobe, [http://www.planiglobe.com](http://www.planiglobe.com/), CC BY 2.0” in the image credit after the caption. (<http://www.planiglobe.com/?lang=enl>)
* Natural Earth - All maps are public domain. (<http://www.naturalearthdata.com/about/terms-of-use/>).

**Answer:** To clarify, the map included in our study (Figure# 1) was created using Tableau 2021.2, a software known for utilizing publicly accessible maps. Specifically, the global map used in our analysis is obtained directly from OpenStreetMap® (OSM). We would like to emphasize that OpenStreetMap® is open data and is licensed under the Open Data Commons Open Database License (ODbL) by the OpenStreetMap Foundation (OSMF). Please see: <https://www.openstreetmap.org/copyright>

According to the licensing terms of OpenStreetMap, users are granted the freedom to copy, distribute, transmit, and adapt the data, as long as proper credit is given to OpenStreetMap and its contributors. In the case of our article, we acknowledge and credit OpenStreetMap and its contributors for the use of their data. Furthermore, we comply with the requirements of the Creative Commons Attribution-Share Alike 2.0 license (CC BY-SA 2.0), which governs the distribution of derivative works based on the OpenStreetMap data.

We understand the importance of adhering to copyright policies and ensuring that all materials used in our article comply with the necessary licenses. Based on the information provided above, we believe that our usage of the map in question aligns with the requirements set by PLOS for publication under the Creative Commons Attribution 4.0 International (CC BY 2.0) license.

We have also included some information about that in the Methods section as follows “The map illustrating the distribution of YLL (Years of Life Lost) was created using Tableau 2021.2 software, with a base map layer sourced from OpenStreetMap®, an open data platform”.

3. Please review your reference list to ensure that it is complete and correct. If you have cited papers that have been retracted, please include the rationale for doing so in the manuscript text, or remove these references and replace them with relevant current references. Any changes to the reference list should be mentioned in the rebuttal letter that accompanies your revised manuscript. If you need to cite a retracted article, indicate the article’s retracted status in the References list and also include a citation and full reference for the retraction notice.

**Answer:** We have revised the references section again. We did not need to implement new changes in your references, and just added 2 new references.

**Reviewer #1:**

Figures/Tables:

1. Figure 1 does not currently make sense with ‘Unisex’ presented alongside ‘Male’ and ‘Female’. If only looking at the axis and not the values, it may be assumed that ‘Unisex’ would be showing a combined total for the two sexes presented. I do not think that this Figure should be presented as is, without country-specific information that makes clear why the ‘Unisex’ values are so different to the ‘Male’ and ‘Female’ values. The Supplementary Figures provide more useful information, in separating countries that do/don’t have data by sex clearly. A more meaningful ‘Figure 1’ could be to present an age-standardised YLL rate for all countries (separating countries by sex where available, as currently done in the supplementary figures), or show an aggregate of sex, for each life cycle stage (i.e. grouping all countries).

**Answer:** "The figure has been updated based on the reviewer's suggestions and now represents the age-standardized YLL rate for all countries, categorized by sex and life cycle stage. Please note that this revised figure is now identified as **Figure # 2**. All the information by country in detail is only provided in the supplementary tables.

2. Please state the currency of budgets presented in Table 1.

**Answer:** The currency was already expressed in the title of the figure (in brackets). However, we also added the currency in the manuscript as a part of the description of the results of table 1.

Methods

3. The economic analysis is an interesting additional assessment of country-specific performance, and potential estimate for future pandemic planning. However, there are limitations that have not been addressed. Utility weights from the UK have been applied (from the EQ-5D, as stated in the cited study, reference 18: Claxton et al., though this should be stated more clearly in the present paper also), but these are highly unlikely to be accurate for all 49 countries. Secondly, health valuation has been found to vary greatly between populations when using the EQ-5D utility measure (see for discussions: Gerlinger, C., Bamber, L., Leverkus, F. et al. Comparing the EQ-5D-5L utility index based on value sets of different countries: impact on the interpretation of clinical study results. BMC Res Notes 12, 18 (2019); and: Roudijk B, Donders ART, Stalmeier PFM; Cultural Values Group. Cultural Values: Can They Explain Differences in Health Utilities between Countries? Med Decis Making. 2019 Jul;39(5):605-616). This limitation, and the lack of cross-walking or use of country-specific values for quality of life should be clearly addressed in the discussion.

**Answer:** The economic analysis provided in our study offers valuable insights into country-specific performance and serves as a potential estimate for future pandemic planning. However, it is important to acknowledge and address the limitations that you have been identified. One such limitation is certainly as you said the use of utility weights from the UK, as indicated in the cited study by Claxton et al. (reference 18)..We agree that applying these weights to all 49 countries included in our study is highly unlikely to accurately represent the diverse populations across those countries. However, that is a common challenge in this kind of analysis.

Furthermore, previous research has demonstrated significant variations in health valuation when using the EQ-5D utility measure, as discussed in the studies by Gerlinger et al. (reference 33) and Roudijk et al. (reference 34). These variations highlight the importance of considering country-specific values for quality of life, or at least acknowledging the potential impact of using a single set of values across multiple countries. This limitation, along with the absence of cross-walking or the use of country-specific values, should be clearly addressed in the discussion section.

The suggestions are clarified, and the following text is added to the discussion section,

*“**In addition, a limitation of our study with respect to estimating the cost-effective budget for national COVID-19 management relates to the extrapolation of the UK quality of life weights to all other countries in the study.* *Although the ideal is to use the specific values of each country, almost none of these have them calculated. Obtaining them presents psychometrics and clinimetrics challenges that do not yet have a comprehensive solution and are still being studied by health economists (34, 35).”*

Two new references are included:

*34.	Gerlinger C, Bamber L, Leverkus F, Schwenke C, Haberland C, Schmidt G, et al. Comparing the EQ-5D-5L utility index based on value sets of different countries: impact on the interpretation of clinical study results. BMC Res Notes. 2019;12(1):18.*

*35.	Roudijk B, Donders R, Stalmeier P, Luo N, Viney R, Andrade M, et al. Cultural values: can they explain differences in health utilities between countries? Med Decis Mak. 2019;39(5):605–16.*

By explicitly acknowledging these limitations and providing references to relevant research, we aim to convince the reader that despite these shortcomings, our study still offers valuable insights and serves as a significant contribution to the field.

4. Please state whether discounting has been applied to YLL calculations (it is inferred that YLLs are not discounted in this paper, but this should be stated/justified). Note that UK recommendations are to discount costs and benefits at 3.5% per year (Claxton et al.) Presenting discounted results in the supplement would be more thorough and provide this option for readers.

**Answer:** In response to the question about discounting in our calculations of Years of Life Lost (YLL), it is important to clarify that discounting was not applied in our study, and there is a specific justification for this choice. The application of discounting is typically recommended when conducting health economic evaluations, such as cost-effectiveness or cost-utility analyses, where the objective is to standardize costs and health outcomes to a common base year for comparison across different health technologies.

However, in our study, we are not operating within the framework of a health technology assessment, and our aim is not to compare different health technologies. Instead, we analyze each year independently, and therefore the interpretation of results does not require the application of discounting. The results presented in the paper reflect this approach and are correct in the context of our study.

For more information on the relevance of the applicability of the discount rate, we share the following literature:

- Drummond M, Sculpher M, Claxton K, Stoddart G, Torrance G. Methods for the economic evaluation of health care programmes. 4th ed. Oxford: Oxford University Press; 2015.
- National Institute for Health and Care Excellence. Discounting. Londres; 2020.
- Claxton K, Paulden M, Gravelle H, Brouwer W, Culyer A. Discounting and decision making in the economic evaluation of health-care technologies. Health Econ. 2011;20(1):2–15.
- John J, Koerber F, Schad M. Differential discounting in the economic evaluation of healthcare programs. Cost Eff Resour Alloc. 2019;17(1):29.
- Williams A, Rojanasarot S, McGovern A, Kumar A. A systematic review of discounting in national health economic evaluation guidelines: healthcare value implications. J Comp Eff Res. 2022.
- Gravelle H, Smith D. Discounting for health effects in cost-benefit and cost-effectiveness analysis. Health Econ. 2001 Oct;10(7):587–99.
- Lomas J, Claxton K, Ochalek J. Accounting for country- and time-specific values in the economic evaluation of health-related projects relevant to low- and middle-income countries. Health Policy Plan. 2022;37(1):45–54.

Results

5. Two statements are made regarding differences by sex, which are currently conflicting. On page 9, line 156-157, it is stated that a higher burden was found for males by all life cycle stages. However, on page 13, line 207, it is stated that no systematic differences in YLLs were found by gender. Please clarify these statements.

**Answer:** Clarified as suggested (eliminating the first interpretation), thanks for the recommendation.

Discussion

6. The main limitations of the study are well addressed in the discussion, particularly with regard to likely underreporting of COVID-19 deaths in lower income countries due to poor health information system capacity/strength. However, given this limitation, have the authors considered calculating an uncertainty range for each country’s YLL estimate, perhaps with a measure of health information system strength being used to assign different levels of uncertainty in presented YLL? If this is not possible, the authors should consider presenting an additional sub-analysis, grouping countries by a proxy of health information system strength (e.g. low/mid/high income countries), to avoid inaccurate comparisons being made between countries with likely high variation in the accuracy of reported deaths.

**Answer:** Thank you for your suggestion. We appreciate your concern regarding the potential underreporting of COVID-19 deaths in lower income countries and the need to consider uncertainties in the estimates of Years of Life Lost (YLL) for each country. However, we believe that exploring this aspect falls outside the scope of our current study.

Calculating an uncertainty range for each country's YLL estimate would require a different methodological approach and additional data sources, specifically related to health information system strength. Unfortunately, we do not have access to such data in our study.

Nevertheless, we fully acknowledge the importance of the issue you raised, and we have addressed it in detail in the discussion section of our paper. We highlight the potential limitations stemming from underreporting in lower income countries and the potential variations in the accuracy of reported deaths between countries. By providing this contextual information, we aim to ensure that readers are aware of these limitations and can interpret the results appropriately.

Once again, we appreciate your valuable input and the opportunity to address this concern.

**Reviewer #2**

Abstract

The abstract should provide a more detailed explanation of the population, sampling, procedure of the Years of Life Lost (YLL), and statistical methods used rather than merely stating the aim of the study. Additionally, the duration of the study should not be repeated in the abstract. Instead of saying, "We did not detect differences," it would be more appropriate to state "We found no difference."

**Answer:** The abstract is adjusted according to the suggested recommendations.

Main Text

It is recommended to adhere to the reporting guidelines outlined in the Strengthening the Reporting of Observational Studies in Epidemiology (STROBE) statement. Following these guidelines will enhance the reporting of the analysis section and the measurement section of YLL and QALY. Moreover, it is important to provide recommendations regarding the policy-level implications of this study in the current stage of the pandemic or for future pandemics.

The results should be reported under appropriate subheadings, which will reflect the research question raised in the introduction of the study.

**Answer:** The 22 items in the STROBE list (observational studies) have been reviewed and the article has been adjusted.
