# Supplementary material for: Years of life lost to COVID-19 in 49 countries: A gender- and life cycle-based analysis of the first two years of the pandemic
Source: PLOS Glob Public Health. 2023 Sep 18;3(9):e0002172. doi: 10.1371/journal.pgph.0002172 (PMC10506703; doi:10.1371/journal.pgph.0002172)
Supplement: Attachment — Submitted filename: R&R comments to reviewersAugust14.docx [file pgph.0002172.s019.docx]

**Manuscript Title: Years of life lost to COVID-19 in 49 countries: A gender- and life cycle-based analysis of the first two years of the pandemic.**

**Response to Editor and Reviewers**

**Response to editors:** We would like to express our deepest gratitude to you for taking over our manuscript from the previous editor and continuing the review process seamlessly.

We would also like to extend our thanks to the prior editor for their initial efforts, even though they are no longer part of the process.

Lastly, we are profoundly thankful for the support from the journal Plos Global Public Health. We firmly believe that, due to your invaluable feedback and insights, our article has greatly improved. We're grateful for the opportunity to contribute to the academic community through your esteemed platform.

**Journal requirements**

**1.** Please review your reference list to ensure that it is complete and correct. If you have cited papers that have been retracted, please include the rationale for doing so in the manuscript text or remove these references and replace them with relevant current references. Any changes to the reference list should be mentioned in the rebuttal letter that accompanies your revised manuscript. If you need to cite a retracted article, indicate the article’s retracted status in the References list and include a citation and full reference for the retraction notice.

**Response:** We have thoroughly reviewed our reference list and can confirm that there are no retracted articles cited. We appreciate your diligence and have ensured the accuracy of all references in the manuscript.

**2.** We do not publish any copyright or trademark symbols that usually accompany proprietary names, e.g. (R), (C), or TM (e.g. next to drug or reagent names). Please remove all instances of trademark/copyright symbols throughout the text, including ® on pages 6.

**Response:** We have carefully reviewed and removed all copyright and trademark symbols as you instructed, including the one on page 6. Thank you for pointing this out.

**Reviewer(s)' Comments to Author:**

Please use the space provided to explain your answers to the questions above. You may also include additional comments for the author, including concerns about dual publication, research ethics, or publication ethics. (Please upload your review as an attachment if it exceeds 20,000 characters)

**Reviewer #1:** Thank you to the authors for addressing my comments. I believe this work is suitable for publication (given some final comments below).

1. In relation to my original question regarding currency: the currency is shown in the table heading and, in the text, as 'dollars'. This should be more clearly specified, as done in the referenced study by Woods et al., which notes that CETs are presented in purchasing power parity adjusted US dollars (USD).

**Response:** We have adjusted the title of Table 1 to address your suggestion regarding currency clarification. We've now specified in both the table heading and the text that the currency is denoted as 'dollars'. Following the example from the referenced study by Woods et al., we have clarified that the CETs are presented in purchasing power parity adjusted US dollars (USD). We appreciate your feedback and the opportunity to provide this clarification.

2. On page 10, line 225: the authors state that deaths and YLLs combined for older adults adds to 947.0%. There is also a closed bracket after this number, with no open bracket.

**Response:** Thank you for pointing out this discrepancy. Indeed, this was an oversight during data transfer. We have now corrected the number on page 10, line 225. Furthermore, to ensure there are no other typographical errors, we have meticulously reviewed all the numbers throughout the manuscript and can confirm their accuracy. The extraneous closed bracket has also been addressed. We appreciate your keen observation and the opportunity to rectify the error.

3. Figure 2 now makes more sense. However, in the text (page 6 line 153-155) the authors state that this figure shows data from all 49 countries (with the figure title also stating 'all studies countries'). This figure presents results by sex, so would seemingly only include the countries that had data available by sex (36 countries, per the Methods section). Please update the text and figure title as needed.

**Response:** Thank you for your observation regarding Figure 2. You are correct. We have now clarified both in the text and in the title of Figure 2 that the data presented pertains only to the 36 countries for which sex-disaggregated data was available. We appreciate your attention to detail and have made the necessary adjustments to ensure accuracy and clarity.

Thank you, Reviewer 1, for all your invaluable feedback and support throughout this review process. Your insights and detailed observations have greatly enriched our work, and we truly appreciate your dedication to ensuring the quality and accuracy of our manuscript.

**Reviewer #2:** No comments at this stage of revision.

**Response:** Reviewer #2: We appreciate your time and efforts in reviewing our manuscript. Thank you for your thorough examination, and we're grateful for your confirmation that there are no comments at this stage of revision. Your feedback throughout has been instrumental to our work.
